# Measuring Stress and Perceptions for a Virtual Reality–Based Pericardiocentesis Procedure Simulation for Medical Training: Usability Study

**DOI:** 10.2196/68515

**Published:** 2025-05-07

**Authors:** Alberto Rubio-López, Rodrigo García Carmona, Laura Zarandieta Román, Alejandro Rubio Navas, Angel González-Pinto, Pablo Cardinal-Fernández

**Affiliations:** 1 Intensive Care Unit, Hospital Universitario HM Montepríncipe, HM Hospitales, Avda Montepríncipe, Boadilla del Monte, Madrid, 28660, Spain, 34 656316182; 2 Facultad de Medicina, Universidad San Pablo CEU, Madrid, Spain; 3 Departamento de Tecnologías de la Información, Escuela Politécnica Superior, Universidad San Pablo CEU, Madrid, Spain; 4 Departamento de Fisiología, Facultad de Biología, Universidad Autónoma de Madrid, Madrid, Spain; 5 Jefe de Servicio. Unidad de Cirugía Cardiaca, Hospital General Universitario Gregorio Marañón, Madrid, Spain; 6 Jefe de Servicio de Cirugía Cardiaca, Hospital Universitario HM Montepríncipe, HM Hospitales, Boadilla del Monte, Madrid, Spain; 7 Intensive Care Unit, Hospital Universitario HM Torrelodones, HM Hospitales, Madrid, Spain; 8 Facultad HM de Ciencias de la Salud, Camilo José Cela University, Villanueva de la Cañada, Madrid, Spain

**Keywords:** VR, pericardiocentesis simulation, usability assessment, heart rate variability, physiological stress, simulator sickness, System Usability Scale, Presence Questionnaire, virtual reality

## Abstract

**Background:**

Virtual reality (VR) is increasingly used in medical education, providing immersive environments for training in high-risk procedures such as pericardiocentesis. This lifesaving procedure requires technical precision and induces cognitive and physiological stress. Evaluating both usability and stress responses in a VR-based pericardiocentesis simulation is essential. Heart rate variability (HRV) serves as an objective stress marker, while prior VR experience may influence usability and stress perception.

**Objectives:**

This study aimed to assess the usability of a VR-based pericardiocentesis simulation, examine the relationship between usability perceptions and physiological stress (HRV), and determine the impact of prior VR experience on usability scores and stress responses.

**Methods:**

A total of 119 final-year medical students participated in a VR pericardiocentesis simulation. Usability was evaluated using the System Usability Scale (SUS), the Post-Study System Usability Questionnaire, the Presence Questionnaire, and the Simulator Sickness Questionnaire. Physiological stress was assessed through HRV parameters, including the root-mean-square of successive differences (rMSSDs), percentage of differences greater than 50 ms (PNN50), low-frequency to high-frequency ratio, and nonlinear HRV indices (SD1/SD2 ratio, Poincaré area). Statistical analyses included descriptive statistics, Spearman correlations, and Mann-Whitney *U* tests to explore relationships between usability, stress, and prior VR experience.

**Results:**

The VR simulation received a mean SUS score of 75.00 (SD 6.41; 95% CI 73.42‐76.58), exceeding the general usability threshold of 68 (*P*=.002). The mean Post-Study System Usability Questionnaire score of 2.92 (SD 1.83; 95% CI 2.55‐3.29) indicated moderate satisfaction, while the mean Presence Questionnaire score of 109.46 (SD 9.12; 95% CI 107.88‐111.04) reflected strong immersion. Simulator sickness symptoms were mild (mean Simulator Sickness Questionnaire score 12.43, SD 15.41; 95% CI 9.28‐15.58), although novice users reported significantly higher nausea levels (*P*=.02). Physiological stress analysis revealed a mean rMSSD of 281.27 (SD 98.99; 95% CI 259.45‐303.09) ms and PNN50 of 56.85% (SD 19.70%; 95% CI 52.23%‐61.47%), indicating moderate autonomic balance. A significant negative correlation was observed between HRV parameters (rMSSD and PNN50) and simulator sickness (*P*=.04; Spearman ρ=−0.23), suggesting that higher physiological stress was associated with increased simulator sickness symptoms. Prior VR experience was linked to higher usability scores (SUS +5.2; 95% CI 3.12-7.28; *P*=.03) and lower simulator sickness symptoms (*P*=.02) but did not significantly affect HRV markers.

**Conclusions:**

VR-based simulations for high-risk medical procedures are effective training tools with high usability (SUS=75) and strong immersion. Simulator sickness correlated with physiological stress, emphasizing the need for design refinements to improve user comfort. Prior VR experience improved usability and reduced simulator sickness but did not significantly impact HRV markers. Future research should refine VR interfaces to balance immersion with minimized cognitive and physical discomfort.

## Introduction

Virtual reality (VR) has rapidly emerged as a transformative tool in medical education. Its ability to simulate high-risk procedures in a controlled and safe environment has made VR an invaluable resource for developing essential clinical skills [1]. One of these procedures, pericardiocentesis—an urgent, lifesaving intervention that involves inserting a needle into the pericardium to drain fluid around the heart—requires not only technical precision but also the ability to remain calm under extreme pressure [2]. In real clinical settings, this combination of skills is critical, and VR simulations offer a safe and repeatable platform for medical students to develop these critical skills without the risks associated with real-life clinical practice.

The immersive nature of VR is especially effective in recreating the cognitive and emotional challenges clinicians face during real-life medical emergencies. However, this immersion can also induce significant stress in users, particularly when simulating high-stress, high-risk procedures such as pericardiocentesis. To ensure the effectiveness of VR simulations in preparing medical students for real-world scenarios, it is crucial to evaluate both the usability of the system and the physiological stress responses it triggers [3]. Heart rate variability (HRV) is a well-established biomarker used to measure stress, providing objective data on how users manage stress in high-pressure situations. Evaluating both aspects is essential to optimize VR-based training and ensure that students are adequately prepared for high-pressure clinical scenarios [4].

For VR to function as an effective educational tool, it must be intuitive and user-friendly. Standard usability scales, such as the System Usability Scale (SUS) [5] and the Post-Study System Usability Questionnaire (PSSUQ) [6], assess how efficiently users interact with the system and whether the technology facilitates or hinders learning. A high usability score indicates a low-cognitive load, allowing learners to focus on the procedural and clinical aspects of the task rather than on navigating the VR environment [7].

Another key factor in effective VR training is presence, defined as the degree to which users feel immersed in the virtual environment. The Presence Questionnaire (PQ) [8] evaluates this aspect by measuring user engagement and perceived realism. A strong sense of presence is essential for simulating real-world clinical pressure, as it encourages learners to respond as they would in an actual medical emergency [9]. Balancing high usability with a strong presence ensures that VR simulations are both accessible and engaging for medical trainees.

Training for high-risk medical procedures involves not only cognitive demands but also physiological stress. Immersive VR simulations have been shown to trigger autonomic nervous system (ANS) responses, which can influence performance, learning, and emotional regulation [10]. HRV is a well-established physiological marker of stress, providing insights into autonomic regulation during demanding tasks [11]. HRV parameters reflect different aspects of autonomic function:

- Parasympathetic (vagal) tone: indicators such as the root-mean-square of successive differences (rMSSD), proportion of NN50 (pNN50), high-frequency (HF) power, and standard deviation 1 (SD1) are associated with relaxation and adaptive stress responses [12].
- Overall ANS oscillations: metrics including SD of NN intervals, low-frequency (LF) power, and standard deviation 2 (SD2) represent the combined influence of both sympathetic and parasympathetic branches of the ANS, indicating autonomic flexibility and stress regulation.
- Sympathovagal balance: the LF/HF ratio and SD2/SD1 ratio indicate the relative dominance of sympathetic (stress-related) and parasympathetic (relaxation-related) activity, where a higher LF/HF ratio suggests greater sympathetic activation.

By analyzing these HRV parameters, researchers can assess how physiological stress responses interact with cognitive and emotional demands in VR-based medical training [13]. However, the extent to which these markers correlate with usability and immersion remains unclear. User experience with VR varies significantly, and prior exposure to VR technology may affect both usability perceptions and stress responses [14]. Medical students with previous VR experience may find the system more intuitive, report higher usability scores, and experience less stress, as they are already familiar with navigating virtual environments. Conversely, students without prior VR exposure may struggle with interaction mechanics, leading to heightened cognitive load and stress, particularly in high-stakes scenarios such as pericardiocentesis. Understanding how prior VR experience influences usability and physiological stress is crucial for designing adaptive VR training programs that accommodate users with varying levels of familiarity.

While previous research has investigated VR usability and stress responses, few studies have explored their relationship, especially in the context of high-risk medical procedures such as pericardiocentesis. Furthermore, the role of prior VR experience in shaping both usability perceptions and physiological adaptation to stress remains poorly understood. This study aimed to fill this gap by examining the interplay between usability, HRV-based stress responses, and prior VR experience in a cohort of final-year medical students undergoing a pericardiocentesis simulation. Findings from this research will provide valuable insights into how VR training environments can be refined to better prepare students for the cognitive and physiological demands of real-world clinical practice.

## Methods

### Participants

A total of 119 final-year medical students were recruited through in-class announcements. During lecture sessions, researchers provided an overview of the study’s objectives, procedures, risks, and benefits, allowing students to ask questions before deciding to enroll. Participation was voluntary, with no financial or material compensation provided. Interested students signed informed consent forms, which outlined confidentiality procedures and withdrawal rights. Recruitment was completed within 1 month. Participants ranged in age from 22 to 45 (mean 24.03, SD 3.4) years, with 78.4% (93/119) identifying as female. The sample was representative of the student body demographics at the institution. Prior VR experience was assessed as a binary variable (yes/no)—participants reported whether they had previously used VR for any purpose. Those with prior VR exposure (31/119, 26.1%) engaged with VR-based applications (eg, gaming, training, and immersive experiences), whereas the remaining 73.9% (88/119) had no prior VR experience.

### Inclusion and Exclusion Criteria

Eligible participants were final-year medical students aged 22‐45 years without uncorrected visual impairments. Students using corrective eyewear (glasses or contact lenses) were eligible. To ensure the validity of HRV measurements and usability assessments, the following exclusion criteria were applied: (1) cardiac medication use (eg, β-blockers) due to potential effects on autonomic regulation; (2) excessive caffeine consumption (>400 mg/day, equivalent to ~4 cups of coffee), as it can alter physiological stress responses; (3) use of sympathomimetic substances (eg, decongestants and stimulants) due to potential HRV interference; (4) history of severe motion sickness, which could bias usability and simulator sickness assessments; and (5) incomplete data or poor-quality HRV recordings, ensuring data integrity. These criteria minimized confounding factors and ensured a controlled evaluation of usability and physiological responses.

### Instruments

#### Usability Scales

After completing the VR simulation, participants electronically completed four validated questionnaires via Microsoft Forms. They accessed the forms on personal devices in a quiet environment to ensure consistent data collection.

- The System Usability Scale is a 10-item, 5-point Likert scale measuring ease of use (0‐100, higher scores=better usability). It includes 2 subscales: SUS-Usability (overall ease of interaction) and SUS-Learning (effort required to learn system use).
- The Post-Study System Usability Questionnaire is a 16-item scale measuring user satisfaction in 3 domains: system usefulness, information quality, and interface quality. Responses are recorded on a 7-point Likert scale, where lower scores indicate higher usability perceptions.
- The Presence Questionnaire is a 24-item scale assessing immersion within the VR environment, with subscales measuring involvement, control, and interface quality. Scores range from 1 (“not at all”) to 7 (“very much”), with higher scores reflecting stronger engagement with the virtual environment.
- The Simulator Sickness Questionnaire (SSQ) evaluates symptoms of simulator sickness across 3 domains: nausea, oculomotor discomfort, and disorientation. Participants rate their symptoms on a 4-point scale (0=“none” to 3=“severe”). The SSQ provides both individual subscale scores and a total sickness score.

The complete versions of these questionnaires are available as MultimediaAppendices 1^4^.

#### HRV Parameters

**Figure 1.**
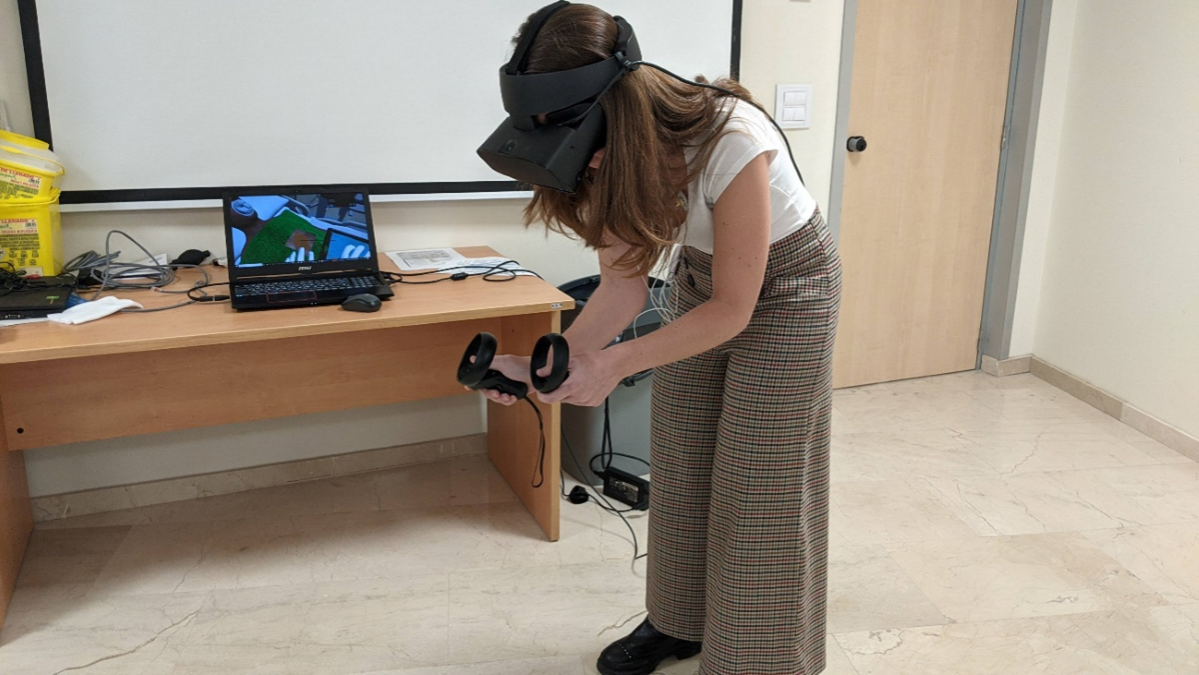
Participant interacting with the virtual reality simulation for pericardiocentesis using Oculus Rift S. The simulation replicates the procedural steps required in a real clinical setting. The session lasted approximately 10 minutes and included real-time physiological stress monitoring via heart rate variability measurements.

HRV was continuously recorded using the Biosignal Plux system (Figure 1). Three torso-mounted electrodes measured heart rate, with data processed via OpenSignals (Plux Wireles Biosignals) software to extract key HRV parameters:

- rMSSD: reflects parasympathetic activity, indicating relaxation.
- PNN50: percentage of successive heart rate intervals that differ by more than 50 ms, reflecting vagal tone.
- LF/HF ratio: indicator of autonomic balance (higher= sympathetic dominance, lower= parasympathetic dominance).
- SD1/SD2 ratio: reflects short-term versus long-term HRV variability, with larger values indicating greater autonomic flexibility.

### VR Simulation

The VR pericardiocentesis simulation was developed in Unity 2021.3.24f1 (Unity Technologies) with the Oculus Integration SDK. The environment included a virtual hospital room, a patient bed, vital signs monitor, and interactive procedural tools.

On top of the bed lies a male patient, and besides him are 2 easily accessible tables with the following instruments: three electrodes, a pressure cuff, a pulse oximeter, an oxygen mask, a box of gloves, a Betadine bottle, forceps with a clean gauze, a surgical cloth, a tray with a syringe, a straight endovascular device, and a bent endovascular device. All these items are interactive and should be used to perform the procedure in the proper order and places.

**Figure 2.**
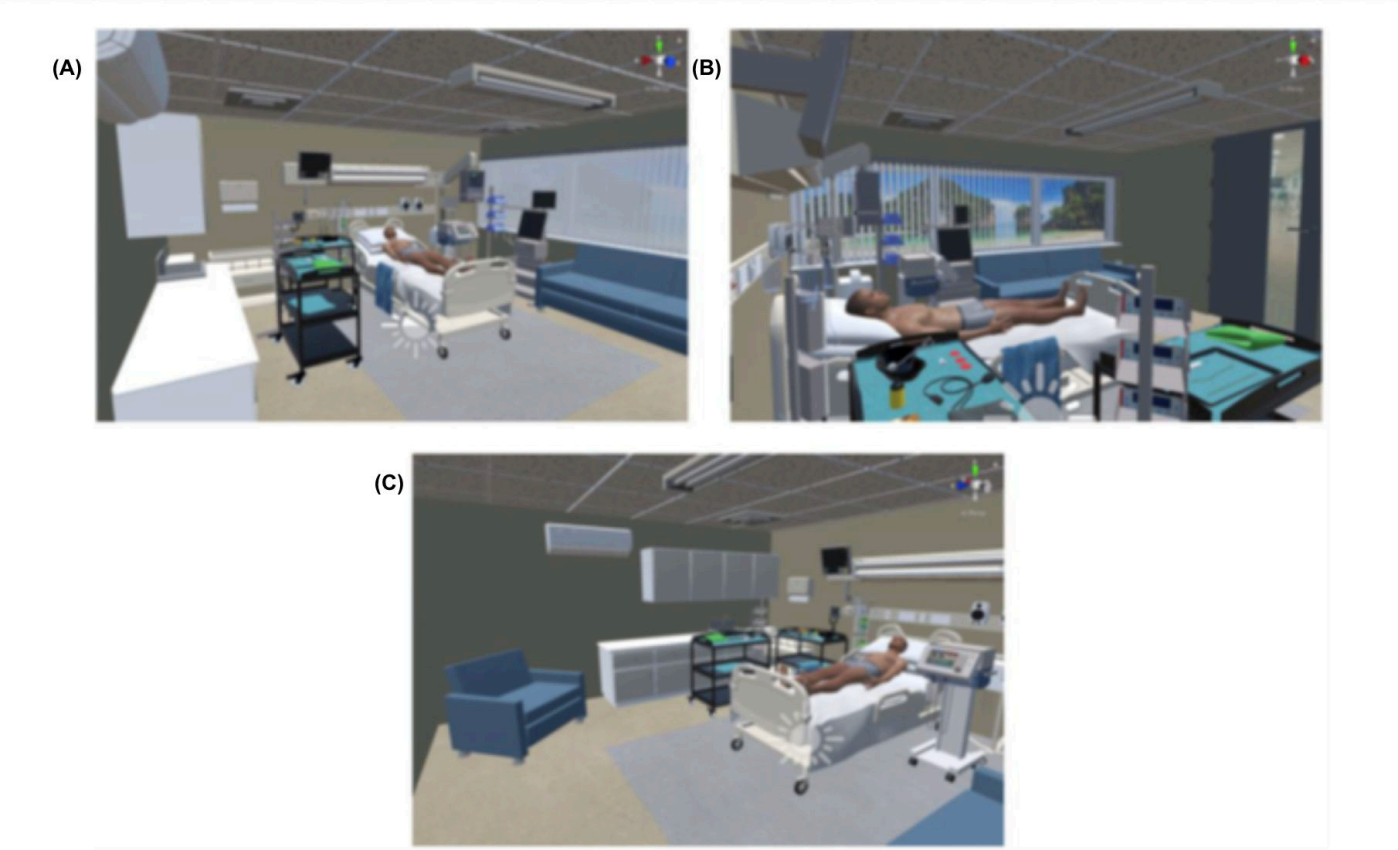
Virtual hospital environment for pericardiocentesis training. The virtual reality simulation presents a high-fidelity virtual hospital room containing a patient bed, vital signs monitor, procedural instruments, and interactive medical equipment necessary for pericardiocentesis. The immersive design was developed using Unity 2021.3.24f1 and aimed to enhance skill acquisition and clinical decision-making among medical students. (A) General view of the simulated room environment. (B) Lateral view of the scenario showing the patient and surrounding procedural equipment. (C) Opposite-side perspective providing a panoramic overview of the full training environment.

The vital signs monitor updates when the pressure cuff, the electrodes, the pulse oximeter, and the oxygen masks are placed on the patient, and when the procedure finishes. The data shown on the monitor are accompanied by a beeping sound, recorded from a real device. The complete setup can be seen in Figure 2.

**Figure 3.**
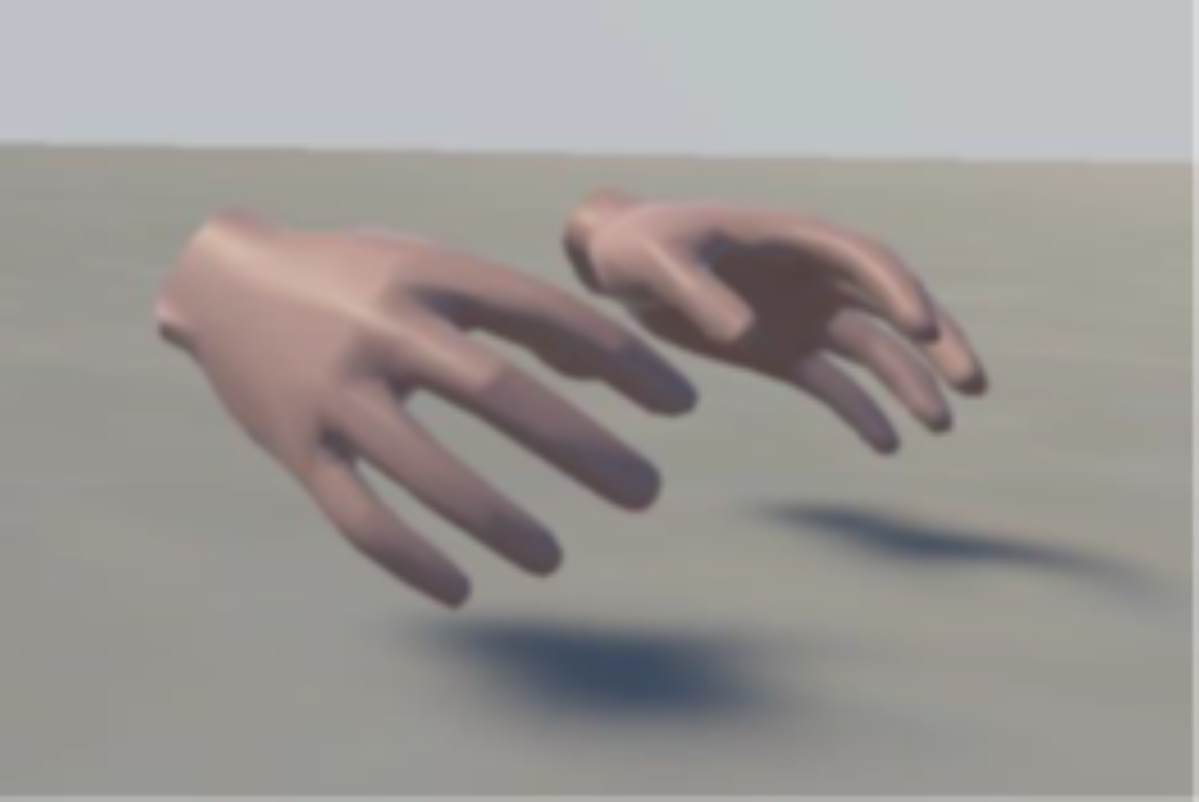
Virtual hand interface used for procedural interactions. The virtual reality system uses diegetic interaction controls, where users interact with virtual elements directly replicating real-world medical tools. The hands, visible within the simulation, dynamically update to include surgical gloves when appropriate. This design eliminates elements not present in the real world such as teleportation mechanics or external heads-up display, ensuring a realistic and immersive training experience.

**Figure 4.**
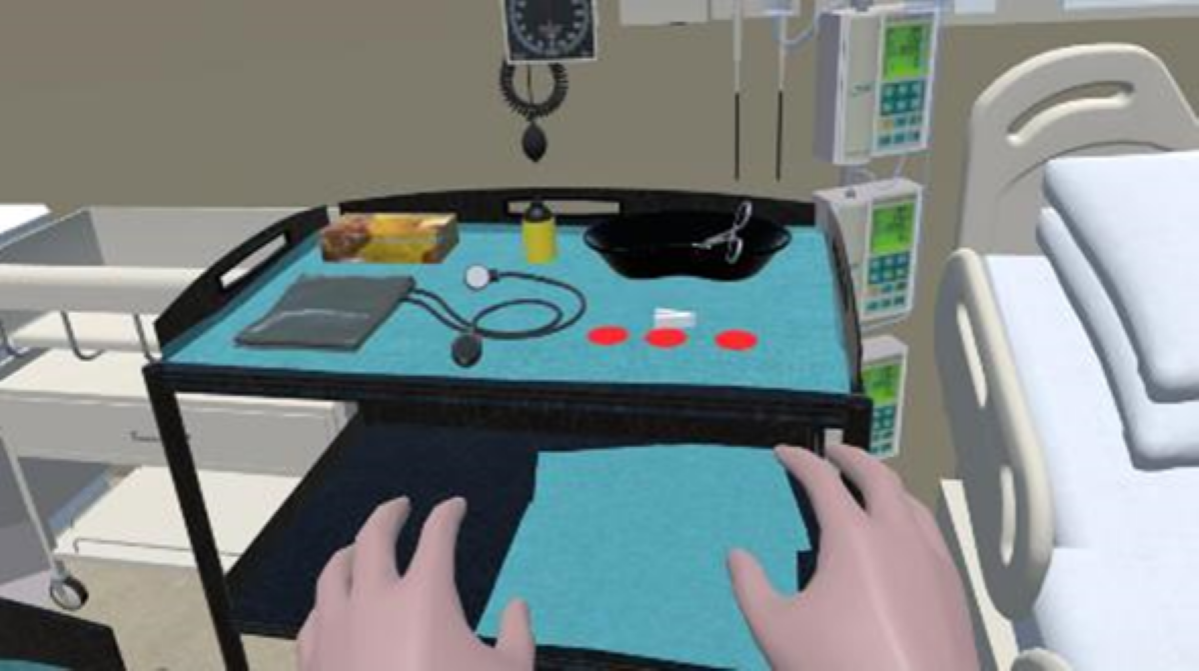
User engagement within the virtual reality (VR) simulation. Still image from a recording of a participant performing pericardiocentesis inside the VR environment. The participant is navigating the simulation using handheld Oculus Rift controllers, interacting with virtual instruments to complete procedural steps. The VR session was part of a controlled study evaluating usability, immersion, and physiological stress responses in final-year medical students.

The user does not see their own body but can see 2 virtual hands (Figure 3), which also show the surgical gloves, if appropriate. All the interactions inside the simulation were diegetic: using virtual elements present in the scenario that replicate real items, without any menu, teleportation mechanism, heads-up display, or other immersion-breaking controls (Figure 4). The user can displace themselves only by direct movement, without any teleportation of stick-controlled movement mechanisms.

The VR simulation was conducted using the Oculus Rift S headset, which was calibrated before each session to ensure accurate tracking and optimal user experience. The calibration process included adjusting the interpupillary distance, ensuring proper headset fit, and setting the default room-scale boundary to prevent tracking errors. Participants were instructed on how to adjust the headset for comfort, ensuring that the lenses were correctly aligned to minimize motion blur and discomfort. Environmental conditions were controlled to ensure consistency across all sessions. The simulation took place in a dedicated laboratory with dim lighting to reduce glare and reflections and a quiet setting to minimize distractions. Participants were allowed limited standing movement within a 3×3 m play area, which was predefined using the Guardian System of the Oculus Rift S to prevent accidental collisions. The temperature was maintained at a comfortable level to avoid physiological stress unrelated to the simulation. Each session lasted approximately 30 minutes, including headset fitting, familiarization with the controls, and the execution of the pericardiocentesis procedure. To ensure consistency across participants, all interactions were standardized, and the experimenter provided identical procedural instructions before each session.

### Procedure

Participants completed the VR pericardiocentesis simulation in three phases:

1. Pre-VR phase: before starting the simulation, participants completed demographic and health behavior questionnaires, and their baseline HRV data were recorded. Participants also reported their prior experience with VR through a binary yes/no response.
2. VR session: throughout the VR simulation, HRV was continuously measured to assess participants’ physiological responses to stress. All tasks were time-stamped to facilitate precise analysis of stress responses during different phases of the simulation
3. Post-VR phase: after completing the simulation, questionnaires were administered on the web using Microsoft Forms to ensure ease of completion and data collection. Completion of the questionnaires required approximately 15 minutes. All responses were automatically anonymized upon submission to protect participant confidentiality.

### Ethical Considerations

The study was approved by the University Hospital research ethics committee (code: 18.12.1339.GHM), following the Declaration of Helsinki. Participants provided written informed consent after a full briefing on study objectives, risks, and withdrawal rights. No financial or material incentives were provided, and all participant data were anonymized.

### Data Analysis

#### Descriptive Statistics

Descriptive analyses were conducted to summarize participants' responses across all questionnaires, including SUS, PSSUQ, PQ, and SSQ, as well as key HRV parameters. Measures of central tendency (mean), dispersion (SD), and distribution (percentiles) were computed to characterize usability perceptions, simulator sickness symptoms, presence levels, and physiological stress markers. These summary statistics provided the foundation for subsequent inferential analyses and helped identify potential differences based on prior VR experience.

#### Reliability Analysis

To evaluate the internal consistency of the instruments used in this study, Cronbach alpha was calculated for the SUS, PSSUQ, PQ, and SSQ scales, including their respective subscales. Reliability estimates provide insight into the coherence of participants’ responses across items within each scale, indicating how well the instruments measure usability, presence, and simulator sickness. Alpha values above 0.8 were interpreted as strong reliability, while values below 0.7 were considered to indicate potential measurement inconsistency. These analyses ensured the robustness of the self-reported measures used throughout the study.

### Statistical Tests

Spearman correlation examined relationships between HRV metrics, usability, and simulator sickness. Mann-Whitney *U* tests compared usability and stress responses between participants with and with no VR experience, while Friedman tests compared overall usability scores across scales. All data were securely stored and anonymized following institutional data protection guidelines. Statistical analyses were performed using IBM SPSS Statistics (version 25.0; IBM Corp), and physiological data were processed using OpenSignals software to ensure accuracy and reliability in HRV analysis.

## Results

### Usability Scores

The usability of the VR simulation was assessed using the SUS, PSSUQ, PQ, and SSQ. The internal reliability of these scales was evaluated using Cronbach α (Table 1). High reliability (α>0.8) was found for SUS (α=0.810) and PSSUQ (α=0.980), confirming their consistency in assessing usability and user satisfaction. Some subscales, such as Nausea Symptoms (SSQ; α=0.472) and Perceived Naturalness (PQ; α=0.608), exhibited lower reliability, likely due to individual variability in motion sickness susceptibility and expectations regarding VR realism.

Despite these variations, the overall reliability of the usability scales remains robust, supporting the validity of the findings. Future studies could refine these subscales or integrate biometric indicators (eg, galvanic skin response and pupil dilation) to improve measurement consistency.

**Table 1.** Reliability analysis of usability and experience assessment scales^a^.

Scale and subscale	Cronbach α values
System Usability Scale	
Overall Usability Score	0.810
Perceived Ease of Use	0.790
Ease of Learning	0.789
Presence Questionnaire	
Overall Presence Score	0.821
Involvement and Control	0.810
Perceived Naturalness of the Environment	0.608
Interface Responsiveness	0.394
Auditory Factors	0.638
Post-Study System Usability Questionnaire	
Overall User Satisfaction	0.980
System Usefulness	0.978
Information Clarity and Accessibility	0.922
Interface Efficiency	0.966
Simulator Sickness Questionnaire	
Overall Simulator Sickness Score	0.782
Nausea Symptoms	0.472
Oculomotor Discomfort	0.800
Disorientation Symptoms	0.342

a Cronbach α values above 0.8 indicate good reliability, while values below 0.7 suggest potential inconsistencies within subscales.

### Usability and Presence Outcomes

#### System Usability Scale

Participants rated the VR simulation as highly usable (mean 75.00, SD 6.41; 95% CI 73.42‐76.58; *P*=.002). This score exceeds the conventional usability benchmark of 68, indicating that users found the system intuitive, efficient, and well suited for medical training. SUS subscales (Perceived Ease of Use and Ease of Learning) suggest that participants found the interface intuitive and required minimal effort to learn the system, a critical factor in medical training scenarios where cognitive load must remain manageable.

#### Post-Study System Usability Questionnaire

The overall PSSUQ score (mean 2.92, SD 1.83; 95% CI 2.55‐3.29) indicated moderate-to-high user satisfaction. System Usefulness (mean 2.89, SD 2.05; 95% CI 2.41‐3.37) and Information Clarity (mean 2.99, SD 1.70; 95% CI 2.56‐3.42) suggest that the system was perceived as informative and functional, supporting learning engagement.

#### Presence Questionnaire

Participants reported a high sense of immersion (mean 109.46, SD 9.12). Involvement and Control scored high (mean 55.24, SD 7.46), highlighting realistic and interactive elements. However, Interface Responsiveness (mean 21.40, SD 5.16) was rated lower, indicating areas for improvement in system feedback and response times.

#### Simulator Sickness Questionnaire

Symptoms of simulator sickness were mild (mean 12.43, SD 15.41; 95% CI: 9.28‐15.58), primarily affecting Oculomotor Discomfort (mean 2.41, SD 3.12; 95% CI 1.84‐2.98), and Nausea Symptoms (mean 1.24, SD 1.66; 95% CI 0.94‐1.54). Participants with prior VR experience reported significantly lower nausea symptoms (*P*=.02; 95% CI 2.14‐5.32), reinforcing the importance of adaptation to immersive environments. The short VR session duration (10 minutes) likely contributed to the low incidence of sickness, aligning with best practices in VR exposure time management.

### VR Experience and Stress Measurement

**Figure 5.**
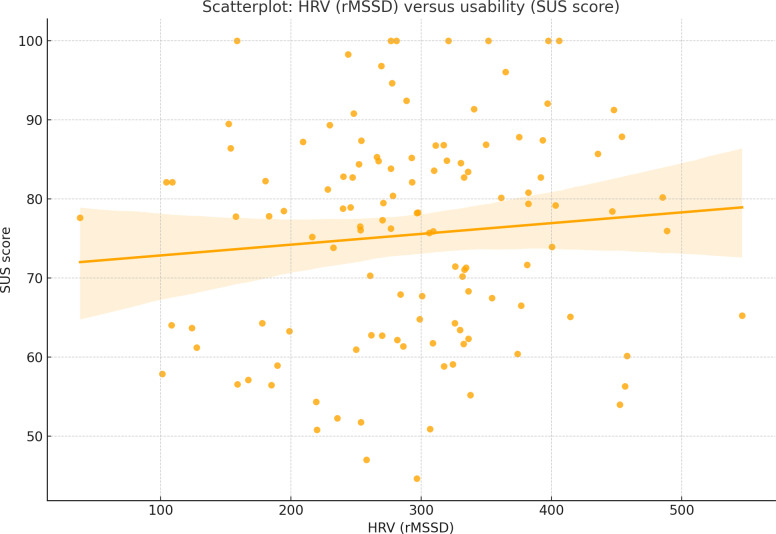
Scatterplot: HRV (PNN50) versus Simulator Sickness. This scatterplot illustrates the correlation between HRV (PNN50) and Simulator Sickness scores, showing a negative trend where higher parasympathetic activity is associated with lower simulator sickness symptoms. HRV: heart rate variability; rMSSD: root-mean-square of successive differences; SUS: System Usability Scale.

Correlational analysis examined physiological stress (HRV) and simulator sickness symptoms (Figure 5):

- rMSSD (mean 281.27 ms, SD 98.99; 95% CI 259.45‐303.09) was negatively correlated with nausea symptoms (*P*=.04), suggesting that greater autonomic relaxation was linked to lower simulator sickness.
- PNN50 (mean 56.85%, SD 19.70; 95% CI 52.23‐61.47) also showed a negative correlation with nausea and oculomotor discomfort (*P*=.04; Spearman ρ=−0.23).
- LF/HF ratio (mean 0.88, SD 0.47) indicated a balanced autonomic state, but no significant correlations were found between LF/HF and usability or presence scores.
- SD1/SD2 and Poincaré area did not correlate significantly with usability or immersion, suggesting that HRV stress markers were more related to physical discomfort than usability perceptions.

**Figure 6.**
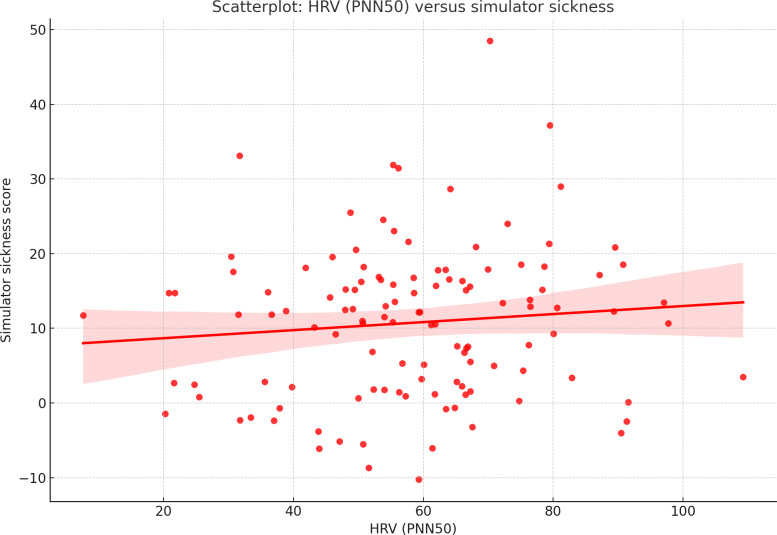
Scatterplot: HRV (root-mean-square of successive differences [rMSSD]) versus usability (System Usability Scale score). This scatterplot shows the lack of significant correlation between HRV (rMSSD) and usability scores, supporting the finding that physiological stress does not strongly influence usability perceptions. HRV: heart rate variability; PNN50: proportion of successive heartbeat intervals differing by more than 50 ms.

HRV analysis revealed that higher parasympathetic activity was associated with lower simulator sickness symptoms. However, HRV metrics did not significantly correlate with usability or presence scores, suggesting that physiological stress responses were more related to physical discomfort than to system usability. No significant correlations were found between HRV (rMSSD) and usability scores, as shown in Figure 6.

### Influence of Prior VR Experience on Results

Participants with prior VR experience reported lower simulator sickness and higher usability perceptions (Table 2):

- Simulator sickness: novice users reported significantly higher nausea symptoms (*U*=997.0; *P*=.02) and oculomotor discomfort (*U*=1047.0; *P*=.045) than experienced users.
- SUS score: VR-experienced participants rated usability significantly higher (+5.2 points; *P*=.03; 95% CI 3.12‐7.28), indicating greater ease of use and reduced cognitive load.
- PQ score: no significant differences in overall presence scores were found, but the Naturalness subscale showed borderline significance (*U*=1088.0; *P*=.09), with experienced users reporting slightly higher realism perception.
- HRV metrics: no significant differences in HRV stress markers between VR-experienced and VR-naive participants, suggesting that prior exposure to VR does not substantially alter physiological stress responses during high-risk simulations.

Prior VR experience was linked to lower levels of simulator sickness but did not significantly affect HRV stress indicators, suggesting that the system was user-friendly across different experience levels.

**Table 2.** Mann-Whitney *U* test comparing participants with and with no prior virtual reality experience^a^.

Outcome measure	No VR^b^ experience (n=88), mean rank	VR experience (n=31), mean rank	*U*	*Z*	*P* value	Significance
SSQ^c^: Nausea	64.17	48.16	997.0	–2.364	.02	Significant
SSQ: Oculomotor	63.60	49.77	1047.0	–2.003	.045	Significant
SSQ: Disorientation	61.94	54.50	1193.5	–1.248	.21	Not significant
SUS^d^: Usability	58.88	63.18	1072.0	–1.770	.08	Marginal
SUS: Learnability	58.03	65.58	1191.0	–1.053	.29	Not significant
PQ^e^: Naturalness	56.86	68.90	1088.0	–1.686	.09	Marginal
HRV^f^: rMSSD^g^	61.14	56.76	1263.5	–0.608	.54	Not significant

a Statistical comparisons were conducted using the Mann-Whitney *U* test. “Marginal” refers to trends toward significance (.05≤*P*<.10). Higher SSQ scores indicate greater simulator sickness and higher SUS scores reflect better usability perceptions.

b VR: virtual reality.

c SSQ: Simulator Sickness Questionnaire.

d SUS: System Usability Scale.

e PQ: Presence Questionnaire.

f HRV: heart rate variability.

g rMSSD: root-mean-square of successive difference.

## Discussion

### Principal Findings

The usability assessments revealed that participants rated the VR system as highly usable, with a mean SUS score of 75 (SD 6.41, 95% CI 73.42-76.58; *P*=.002), exceeding the commonly used benchmark of 68. This indicates that the system was well designed and accessible, allowing users to interact with it effectively. Both novice and experienced users provided comparable usability ratings, suggesting that the reliance on direct movement and the absence of nondiegetic interactions eliminated the need for prior VR navigation experience. These findings align with previous research emphasizing the importance of designing VR systems that are intuitive and inclusive, ensuring accessibility across users with varying levels of expertise [15,16]. Moreover, usability-focused approaches tailored to novice users have been recommended, highlighting the importance of gradual adaptation strategies to facilitate immersion and reduce initial cognitive load [17].

Although usability scores were high, simulator sickness remained a challenge, particularly for novice users, who reported significantly greater nausea and oculomotor discomfort than experienced users. These results are consistent with prior studies showing that previous VR exposure reduces discomfort, likely due to increased adaptation to immersive environments [18]. Adaptation mechanisms such as shorter initial exposure sessions or gradual immersion through simplified VR experiences could facilitate acclimatization for first-time users [19]. The 10-minute session duration may have contributed to minimizing simulator sickness symptoms, as shorter exposure times are recommended to optimize user comfort.

Although simulator sickness was more pronounced among novices, this study did not track symptom progression over time, limiting the ability to assess whether participants acclimated during the session. Research suggests that VR-induced discomfort often declines with repeated exposure as users adapt to the sensorimotor feedback of the environment [20]. Motion sickness symptoms typically peak within the first few minutes of VR exposure and gradually subside, particularly with repeated use. However, because symptoms were measured only at the end of the session, it remains unclear whether adaptation occurred during the simulation itself.

The high SUS score of 75 suggests that participants found the VR system not only intuitive but also effective for training. Previous research evaluating medical VR applications has reported lower initial usability ratings, which tend to improve with repeated exposure. In contrast, the high usability scores observed in this study suggest that the system was well optimized for first-time users, reducing potential barriers to interaction. The Perceived Ease of Use subscale further supports this, indicating that while users found the system easy to navigate, some effort was required to learn the procedural steps. The PSSUQ results, reflecting moderate user satisfaction, are consistent with prior studies evaluating VR-based procedural training, reinforcing the system’s effectiveness for medical education.

The reliability analysis of usability and experience assessment scales confirmed that SUS and PSSUQ demonstrated strong internal consistency. However, some subscales, such as the Nausea Symptoms subscale of the SSQ and the Perceived Naturalness subscale of the PQ, had lower-than-expected reliability. Several factors may account for this variability. Simulator sickness symptoms can vary widely across individuals, influenced by differences in motion sensitivity, vestibular adaptation, and prior VR exposure. Similarly, expectations regarding VR realism may have affected the Perceived Naturalness subscale, particularly among experienced users, who may have had higher expectations for environmental fidelity. While these lower reliability values indicate variability in participant responses, they do not necessarily invalidate the subscales.

The PQ scores indicated a strong sense of immersion among participants, with no significant differences between novice and experienced users, except for a borderline significant difference in the Perceived Naturalness subscale. This suggests that while experienced users may perceive VR environments as more natural, both groups experienced a high level of presence [21]. Maintaining a strong sense of presence is essential in medical simulations, as immersive learning enhances procedural training and stress management skills. Prior studies suggest that enhancing interactivity, improving haptic feedback, and refining visual realism could further increase presence scores in VR-based medical training.

The analysis of HRV data revealed no significant correlations between physiological stress and usability or presence scores. This suggests that participants’ perceptions of usability were primarily cognitive, influenced by their interaction with the interface rather than underlying physiological stress responses. These findings are consistent with previous literature indicating that usability is predominantly shaped by system design and functionality rather than physiological arousal [22]. However, the absence of correlation between HRV and usability or presence does not preclude interactions between cognitive and physiological factors. Several potential mediators or confounding variables could explain this finding. Individual stress tolerance, task difficulty, cognitive load, and personal coping strategies may have influenced HRV responses in ways not fully captured by usability assessments [23]. For instance, some participants may have experienced heightened physiological stress without perceiving usability issues, while others may have found the interface challenging but did not exhibit strong physiological responses. Prior research indicates that task complexity and prior clinical experience can modulate physiological stress responses in simulation-based training [24].

HRV metrics are also influenced by various physiological and psychological factors beyond stress, including baseline autonomic function, fatigue, and attentional demands. Given that VR simulations inherently require motor adaptation and spatial awareness adjustments, some users may have exhibited physiological responses unrelated to usability perceptions. These findings highlight the importance of multidimensional stress assessment approaches that incorporate both HRV and subjective stress measures (eg, NASA-TLX, perceived workload scales) to better capture cognitive and emotional load during VR training.

Interestingly, HRV parameters did not significantly correlate with presence, suggesting that stress responses do not strongly influence immersion in virtual environments. Instead, presence appears to be more dependent on design quality, realism, and interactivity than physiological arousal [25]. These findings align with prior research indicating that immersion in VR is primarily driven by environmental realism and user engagement rather than stress levels [26]. However, subtle interactions between physiological responses and presence may still exist, potentially influenced by individual factors such as trait anxiety or cognitive flexibility. Future research should explore these relationships further.

Recent studies have demonstrated that VR-based stress management interventions, including adaptive exposure and biofeedback integration, can enhance stress resilience and user engagement [27]. The use of real-time physiological feedback, such as HRV-guided VR adjustments, has been proposed as a strategy to optimize stress regulation in immersive environments. Given that stress responses in medical training simulations are complex and influenced by multiple factors, incorporating similar adaptive VR mechanisms in future iterations of medical VR training tools could improve user experience, reduce discomfort, and enhance long-term skill retention [4].

The results demonstrated significant differences in simulator sickness symptoms between participants with and with no prior VR experience. Novice users reported significantly higher levels of nausea and oculomotor discomfort, reinforcing the idea that familiarity with immersive environments helps mitigate the physical discomfort associated with VR [28]. These findings align with prior studies suggesting that repeated VR exposure can lead to adaptation, gradually reducing the severity of simulator sickness symptoms over time [29]. This adaptation effect highlights the need for tailored protocols for novice users, particularly in medical and emergency training contexts, to minimize discomfort and optimize the learning experience.

Despite differences in simulator sickness, there were no significant differences between novice and experienced users in PSSUQ or PQ scores. This suggests that the system was designed to be accessible and intuitive for users regardless of prior VR experience, reinforcing its inclusiveness. The lack of differences in usability scores further supports the idea that first-time users can effectively engage with VR simulations without a steep learning curve [30].

The borderline significance observed in the Perceived Naturalness subscale of the PQ suggests that experienced users may be more attuned to subtle environmental details, contributing to a heightened perception of realism. For novice users, improving environmental naturalness could enhance immersion. While the current simulation featured low-polygon, cartoon-like graphics, incorporating more photorealistic elements may improve engagement, provided that the “uncanny valley” effect is avoided.

### Limitations and Future Directions

This study has several limitations. First, the sample consisted solely of final-year medical students from a single institution, limiting the generalizability of findings to practice clinicians or professionals with different levels of experience. Since clinical expertise influences usability perceptions and physiological stress responses in VR environments, future studies should include more diverse populations across various career stages and medical specialties. Second, usability (SUS and PSSUQ) and simulator sickness (SSQ) were assessed using self-reported measures, which may introduce response biases. External factors such as fatigue or prior VR exposure could have influenced participants’ perceptions. Future research should complement self-reported data with objective behavioral metrics, such as task completion times or interaction errors, to enhance measurement validity. Third, while HRV provided an objective measure of autonomic stress responses, it did not capture cognitive load or psychological stress. The lack of subjective stress assessments (eg, NASA-TLX) and neurocognitive performance measures (eg, reaction times) limits the ability to fully understand the interaction between stress and usability in VR-based procedural training. Fourth, the study’s short VR session (10 minutes) restricted the assessment of long-term adaptation to simulator sickness and stress. Research suggests that symptoms and stress responses diminish with repeated exposure, but this study only measured outcomes postsession. Future studies should track symptom progression at multiple time points to assess adaptation over time. Fifth, the absence of real-world consequences for procedural errors may have reduced perceived urgency and stress. In clinical settings, mistakes during pericardiocentesis can have severe outcomes, whereas errors in VR had no direct impact. Introducing performance-based feedback or simulated consequences could enhance realism and improve training effectiveness. In addition, the low-polygon VR environment, while minimizing cognitive load, may have affected immersion, particularly for users with prior VR experience. Higher-fidelity graphics could enhance realism, although careful design is needed to avoid cognitive overload or the “uncanny valley” effect. Furthermore, a limitation of this study is the binary classification of prior VR experience (yes/no), which does not capture potential differences in familiarity with VR applications, such as gaming, medical simulations, or other interactive experiences. A more granular classification—differentiating casual VR users, gaming-oriented users, and those with professional training in VR environments—could provide deeper insights into how prior exposure influences usability and simulator sickness outcomes.

To address these limitations, future research should include broader participant samples, incorporate both objective and subjective stress measures, evaluate longer VR sessions, and refine simulation realism. Exploring different VR platforms and interaction methods could also inform the best practices for improving usability and immersion in medical training simulations.

### Practical Implications

The findings of this study provide practical insights into optimizing VR-based medical training. Given the high usability of the system (SUS=75), future improvements should prioritize reducing simulator sickness rather than addressing usability concerns. Implementing adaptive exposure strategies—such as shorter initial sessions and gradual complexity increases—could help novice users acclimate, minimizing nausea and oculomotor discomfort. The negative correlation between HRV parameters and simulator sickness symptoms suggests that HRV could be integrated as a real-time monitoring tool to identify users experiencing discomfort. This would allow proactive adjustments, such as pausing sessions or modifying simulation parameters, to enhance user comfort. To maintain usability and realism, VR training should continue using diegetic interactions that mirror real-world clinical procedures, minimizing artificial UI elements that could disrupt immersion. In addition, incorporating performance-based feedback—such as error tracking or consequence-driven scenarios—could reinforce learning and better simulate real-world clinical pressures. By refining exposure strategies, integrating real-time stress monitoring, and enhancing realism, VR-based simulations can provide a more immersive, effective, and user-friendly training experience for medical learners.

### Conclusions

This study suggests that VR-based simulations for high-stress medical procedures, such as pericardiocentesis, offer high usability (SUS=75) and strong immersion, making them effective training tools for users with varying VR experience levels. Physiological stress responses (HRV) correlated more with simulator sickness than with usability or presence, indicating that physical discomfort plays a key role in stress modulation within VR environments.

Prior VR experience was associated with lower simulator sickness symptoms, reinforcing the potential benefits of gradual exposure strategies for first-time users. These findings highlight the complex interaction between usability, stress responses, and prior experience in VR-based medical training.

While these results contribute to understanding VR usability and stress responses, further research is needed to validate these findings in larger and more diverse samples. Future studies should also assess longitudinal effects to determine whether adaptation to simulator sickness occurs over repeated exposures.

## Supplementary material

10.2196/68515 Multimedia Appendix 1 Presence Questionnaire.

10.2196/68515 Multimedia Appendix 2 Post-Study System Usability Questionnaire.

10.2196/68515 Multimedia Appendix 3 System Usability Scale questionnaire used in the study.

10.2196/68515 Multimedia Appendix 4 Simulator Sickness Questionnaire.

10.2196/68515 Multimedia Appendix 5 Summary table of aggregated heart rate variability metrics and usability scores, including subgroup comparisons.

## Abbreviations

ANS: autonomic nervous system
HF: high frequency
HRV: heart rate variability
LF: low frequency
PNN20: proportion of successive heartbeat intervals differing by more than 20 ms
PNN50: proportion of successive heartbeat intervals differing by more than 50 ms
PQ: Presence Questionnaire
PSSUQ: Post-Study System Usability Questionnaire
rMSSD: root-mean-square of successive difference
SD1: standard deviation 1
SD2: standard deviation 2
SSQ: Simulator Sickness Questionnaire
SUS: System Usability Scale
VR: virtual reality
