# Supplementary material for: Measuring Stress and Perceptions for a Virtual Reality–Based Pericardiocentesis Procedure Simulation for Medical Training: Usability Study
Source: JMIR Serious Games. 2025 May 7;13:e68515. doi: 10.2196/68515 (PMC12303137; doi:10.2196/68515)
Supplement: Multimedia Appendix 1 [file games-v13-e68515-s001.pdf]

1-PQ (Presence Questionnaire)

Characterize your experience in the environment, by marking an "X" in the appropriate box of the 7-point scale, in accordance with the question content and descriptive labels. Please consider the entire scale when making your responses, as the intermediate levels may apply. Answer the questions independently in the order that they appear. Do not skip questions or return to a previous question to change your answer.

* Obligatoria

WITH REGARD TO THE EXPERIENCED ENVIRONMENT

1

DNI *

2

How much were you able to control events? *

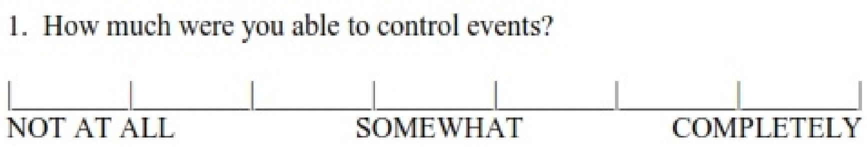
1. How much were you able to control events?

NOT AT ALL SOMEWHAT COMPLETELY

- ◯ 0: NOT AT ALL
- ◯ 1
- ◯ 2
- ◯ 3
- ◯ 4
- ◯ 5
- ◯ 6
- ◯ 7: COMPLETELY

3

How responsive was the environment to actions that you initiated (or performed)? *

2. How responsive was the environment to actions that you initiated (or performed)?

|________|________|________|________|________|________|________|

NOT RESPONSIVE — MODERATELY RESPONSIVE — COMPLETELY RESPONSIVE

- ◯ 0: NOT RESPONSIVE
- ◯ 1
- ◯ 2
- ◯ 3
- ◯ 4
- ◯ 5
- ◯ 6
- ◯ 7: COMPLETELY RESPONSIVE

4

How natural did your interactions with the environment seem? *

3. How natural did your interactions with the environment seem?

|________|________|________|________|________|________|________|

EXTREMELY ARTIFICIAL — BORDERLINE — COMPLETELY NATURAL

- ◯ 0: EXTREMELY ARTIFICIAL
- ◯ 1
- ◯ 2
- ◯ 3
- ◯ 4
- ◯ 5
- ◯ 6
- ◯ 7: COMPLETELY NATURAL

5

How much did the visual aspects of the environment involve you? *

4. How much did the visual aspects of the environment involve you?

|________|________|________|________|________|________|________|

NOT AT ALL SOMEWHAT COMPLETELY

- 0: NOT AT ALL
- 1
- 2
- 3
- 4
- 5
- 6
- 7: COMPLETELY

6

How natural was the mechanism which controlled movement through the environment? *

5. How natural was the mechanism which controlled movement through the environment?

|________|________|________|________|________|________|________|

EXTREMELY ARTIFICIAL BORDERLINE COMPLETELY NATURAL

- 0: EXTREMELY ARTIFICIAL
- 1
- 2
- 3
- 4
- 5
- 6
- 7: COMPLETELY NATURAL

7

How compelling was your sense of objects moving through space? *

6. How compelling was your sense of objects moving through space?

|_______|_______|_______|_______|_______|_______|_______|

NOT AT ALL — MODERATELY COMPELLING — VERY COMPELLING

- 0: NOT AT ALL
- 1
- 2
- 3
- 4
- 5
- 6
- 7: VERY COMPELLING

8

How much did your experiences in the virtual environment seem consistent with your real world experiences? *

7. How much did your experiences in the virtual environment seem consistent with your real world experiences?

|_______|_______|_______|_______|_______|_______|_______|

NOT CONSISTENT — MODERATELY CONSISTENT — VERY CONSISTENT

- 0: NOT CONSISTENT
- 1
- 2
- 3
- 4
- 5
- 6
- 7: VERY CONSISTENT

9

Were you able to anticipate what would happen next in response to the actions that you performed? *

8. Were you able to anticipate what would happen next in response to the actions that you performed?

NOT AT ALL SOMEWHAT COMPLETELY

- 0: NOT AT ALL
- 1
- 2
- 3
- 4
- 5
- 6
- 7: COMPLETELY

10

How completely were you able to actively survey or search the environment using vision? *

9. How completely were you able to actively survey or search the environment using vision?

NOT AT ALL SOMEWHAT COMPLETELY

- 0: NOT AT ALL
- 1
- 2
- 3
- 4
- 5
- 6
- 7: COMPLETELY

11

How compelling was your sense of moving around inside the virtual environment? *

10. How compelling was your sense of moving around inside the virtual environment?

NOT COMPELLING — MODERATELY COMPELLING — VERY COMPELLING

- 0: NOT COMPELLING
- 1
- 2
- 3
- 4
- 5
- 6
- 7: VERY COMPELLING

12

How closely were you able to examine objects? *

11. How closely were you able to examine objects?

NOT AT ALL — PRETTY CLOSELY — VERY CLOSELY

- 0: NOT AT ALL
- 1
- 2
- 3
- 4
- 5
- 6
- 7: VERY CLOSELY

13

How well could you examine objects from multiple viewpoints? *

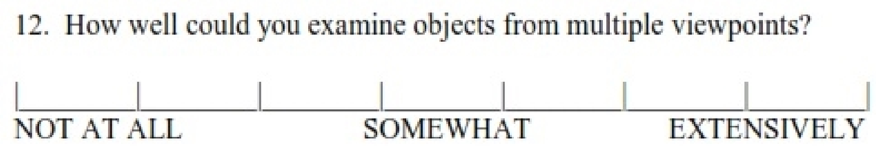
12. How well could you examine objects from multiple viewpoints?

NOT AT ALL SOMEWHAT EXTENSIVELY

- 0: NOT AT ALL
- 1
- 2
- 3
- 4
- 5
- 6
- 7: EXTENSIVELY

14

How involved were you in the virtual environment experience? *

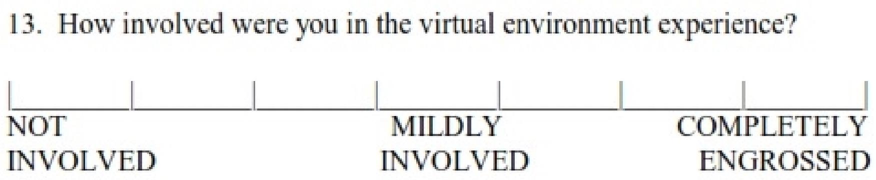
13. How involved were you in the virtual environment experience?

NOT INVOLVED MILDLY INVOLVED COMPLETELY ENGROSSED

- 0: NOT INVOLVED
- 1
- 2
- 3
- 4
- 5
- 6
- 7: COMPLETELY ENGROSSED

15

How much delay did you experience between your actions and expected outcomes? *

14. How much delay did you experience between your actions and expected outcomes?

|________|________|________|________|________|________|________|

NO DELAYS MODERATE DELAYS LONG DELAYS

- 0: NO DELAYS
- 1
- 2
- 3
- 4
- 5
- 6
- 7: LONG DELAYS

16

How quickly did you adjust to the virtual environment experience? *

15. How quickly did you adjust to the virtual environment experience?

|________|________|________|________|________|________|________|

NOT AT ALL SLOWLY LESS THAN ONE MINUTE

- 0: NOT AT ALL
- 1
- 2
- 3
- 4
- 5
- 6
- 7: LESS THAN ONE MINUTE

17

How proficient in moving and interacting with the virtual environment did you feel at the end of the experience? *

16. How proficient in moving and interacting with the virtual environment did you feel at the end of the experience?

|________|________|________|________|________|________|________|

NOT PROFICIENT — REASONABLY PROFICIENT — VERY PROFICIENT

- ◯ 0: NOT PROFICIENT
- ◯ 1
- ◯ 2
- ◯ 3
- ◯ 4
- ◯ 5
- ◯ 6
- ◯ 7: VERY PROFICIENT

18

How much did the visual display quality interfere or distract you from performing assigned tasks or required activities? *

17. How much did the visual display quality interfere or distract you from performing assigned tasks or required activities?

|________|________|________|________|________|________|________|

NOT AT ALL — INTERFERED SOMEWHAT — PREVENTED TASK PERFORMANCE

- ◯ 0: NOT AT ALL
- ◯ 1
- ◯ 2
- ◯ 3
- ◯ 4
- ◯ 5
- ◯ 6
- ◯ 7: PREVENTED TASK PERFORMANCE

19

How much did the control devices interfere with the performance of assigned tasks or with other activities? *

18. How much did the control devices interfere with the performance of assigned tasks or with other activities?

NOT AT ALL — INTERFERED SOMEWHAT — INTERFERED GREATLY

- 0: NOT AT ALL
- 1
- 2
- 3
- 4
- 5
- 6
- 7: INTERFERED GREATLY

20

How well could you concentrate on the assigned tasks or required activities rather than on the mechanisms used to perform those tasks or activities? *

19. How well could you concentrate on the assigned tasks or required activities rather than on the mechanisms used to perform those tasks or activities?

NOT AT ALL — SOMEWHAT — COMPLETELY

- 0: NOT AT ALL
- 1
- 2
- 3
- 4
- 5
- 6
- 7: COMPLETELY

IF THE VIRTUAL ENVIRONMENT INCLUDED SOUNDS:

21

How much did the auditory aspects of the environment involve you? *

20. How much did the auditory aspects of the environment involve you?

|________|________|________|________|________|________|________|

NOT AT ALL SOMEWHAT COMPLETELY

- 0: NOT AT ALL
- 1
- 2
- 3
- 4
- 5
- 6
- 7: COMPLETELY

22

How well could you identify sounds? *

21. How well could you identify sounds?

|_________|_________|_________|_________|_________|_________|_________|

NOT AT ALL SOMEWHAT COMPLETELY

- ◯ 0: NOT AT ALL
- ◯ 1
- ◯ 2
- ◯ 3
- ◯ 4
- ◯ 5
- ◯ 6
- ◯ 7: COMPLETELY

23

How well could you localize sounds? *

22. How well could you localize sounds?

|_________|_________|_________|_________|_________|_________|_________|

NOT AT ALL SOMEWHAT COMPLETELY

- ◯ 0: NOT AT ALL
- ◯ 1
- ◯ 2
- ◯ 3
- ◯ 4
- ◯ 5
- ◯ 6
- ◯ 7: COMPLETELY

IF THE VIRTUAL ENVIRONMENT INCLUDED HAPTIC (SENSE OF TOUCH):

24

How well could you actively survey or search the virtual environment using touch? *

23. How well could you actively survey or search the virtual environment using touch?

|________|________|________|________|________|________|________|
NOT AT ALL SOMEWHAT COMPLETELY

- ◯ 0: NOT AT ALL
- ◯ 1
- ◯ 2
- ◯ 3
- ◯ 4
- ◯ 5
- ◯ 6
- ◯ 7: COMPLETELY

25

How well could you move or manipulate objects in the virtual environment? *

24. How well could you move or manipulate objects in the virtual environment?

|________|________|________|________|________|________|________|
NOT AT ALL SOMEWHAT EXTENSIVELY

- ◯ 0: NOT AT ALL SOMEWHAT
- ◯ 1
- ◯ 2
- ◯ 3
- ◯ 4
- ◯ 5
- ◯ 6
- ◯ 7: EXTENSIVELY

Este contenido no está creado ni respaldado por Microsoft. Los datos que envíe se enviarán al propietario del formulario.

Microsoft Forms
