# Supplementary material for: Measuring Stress and Perceptions for a Virtual Reality–Based Pericardiocentesis Procedure Simulation for Medical Training: Usability Study
Source: JMIR Serious Games. 2025 May 7;13:e68515. doi: 10.2196/68515 (PMC12303137; doi:10.2196/68515)
Supplement: Multimedia Appendix 2 [file games-v13-e68515-s002.pdf]

2-PSSUQ (Post-Study System Usability Questionnaire)

On a scale between Strongly Agree to Strongly Disagree, please rate the following statements:

* Obligatoria

1. DNI *

2. Overall, I am satisfied with how easy it is to use this system (1: Strongly Agree / 7: Strongly Disagree) *

1	2	3	4	5	6	7

3. It was simple to use this system (1: Strongly Agree / 7: Strongly Disagree) *

1	2	3	4	5	6	7

4. I was able to complete the tasks and scenarios quickly using this system. (1: Strongly Agree / 7: Strongly Disagree) *

1	2	3	4	5	6	7

5. I felt comfortable using this system (1: Strongly Agree / 7: Strongly Disagree) *

1	2	3	4	5	6	7

6. It was easy to learn to use this system. (1: Strongly Agree / 7: Strongly Disagree) *

1	2	3	4	5	6	7

7. I believe I could become productive quickly using this system (1: Strongly Agree / 7: Strongly Disagree) *

1	2	3	4	5	6	7

8. The system gave error messages that clearly told me how to fix problems (1: Strongly Agree / 7: Strongly Disagree) *

1	2	3	4	5	6	7

9. Whenever I made a mistake using the system, I could recover easily and quickly (1: Strongly Agree / 7: Strongly Disagree) *

1	2	3	4	5	6	7

10. The information (such as online help, on-screen messages, and other documentation) provided with this system was clear? (1: Strongly Agree / 7: Strongly Disagree) *

1	2	3	4	5	6	7

11. It was easy to find the information I needed (1: Strongly Agree / 7: Strongly Disagree) *

1	2	3	4	5	6	7

12. The information was effective in helping me complete the tasks and scenarios (1: Strongly Agree / 7: Strongly Disagree) *

1	2	3	4	5	6	7

13. The organization of information on the system screens was clear (1: Strongly Agree / 7: Strongly Disagree) *

1	2	3	4	5	6	7

14. The interface of this system was pleasant (1: Strongly Agree / 7: Strongly Disagree) *

1	2	3	4	5	6	7

15. I liked using the interface of this system (1: Strongly Agree / 7: Strongly Disagree) *

1	2	3	4	5	6	7

16. This system has all the functions and capabilities I expect it to have (1: Strongly Agree / 7: Strongly Disagree) *

1	2	3	4	5	6	7

17. Overall, I am satisfied with this system. (1: Strongly Agree / 7: Strongly Disagree) *

1	2	3	4	5	6	7

Este contenido no está creado ni respaldado por Microsoft. Los datos que envíe se enviarán al propietario del formulario.

Microsoft Forms
