# Supplementary material for: Measuring Stress and Perceptions for a Virtual Reality–Based Pericardiocentesis Procedure Simulation for Medical Training: Usability Study
Source: JMIR Serious Games. 2025 May 7;13:e68515. doi: 10.2196/68515 (PMC12303137; doi:10.2196/68515)
Supplement: Multimedia Appendix 3 [file games-v13-e68515-s003.pdf]

3-SUS (System Usability Scale)

On a scale between Strongly Agree to Strongly Disagree, please rate the following statements:

* Obligatoria

1. DNI *

2. I think that I would like to use this system frequently (1: Strongly Disagree / 5: Strongly Agree) *

1	2	3	4	5

3. I found the system unnecessarily complex (1: Strongly Disagree / 5: Strongly Agree) *

1	2	3	4	5

4. I thought the system was easy to use (1: Strongly Disagree / 5: Strongly Agree) *

1	2	3	4	5

5. I think that I would need the support of a technical person to be able to use this system (1: Strongly Disagree / 5: Strongly Agree) *

1	2	3	4	5

6. I found the various functions in this system were well integrated (1: Strongly Disagree / 5: Strongly Agree) *

1	2	3	4	5

7. I thought there was too much inconsistency in this system (1: Strongly Disagree / 5: Strongly Agree) *

1	2	3	4	5

8. I would imagine that most people would learn to use this system very quickly (1: Strongly Disagree / 5: Strongly Agree) *

1	2	3	4	5

9. I found the system very cumbersome to use (1: Strongly Disagree / 5: Strongly Agree) *

1	2	3	4	5

10. I felt very confident using the system (1: Strongly Disagree / 5: Strongly Agree) *

1	2	3	4	5

11. I needed to learn a lot of things before I could get going with this system (1: Strongly Disagree / 5: Strongly Agree) *

1	2	3	4	5

Este contenido no está creado ni respaldado por Microsoft. Los datos que envíe se enviarán al propietario del formulario.

Microsoft Forms
