# Supplementary material for: Measuring Stress and Perceptions for a Virtual Reality–Based Pericardiocentesis Procedure Simulation for Medical Training: Usability Study
Source: JMIR Serious Games. 2025 May 7;13:e68515. doi: 10.2196/68515 (PMC12303137; doi:10.2196/68515)
Supplement: Multimedia Appendix 4 [file games-v13-e68515-s004.pdf]

4-SSQ (Simulator Sickness Questionnaire)

Indicate the degree of current involvement of each of the following symptoms:0: Not affected1: Somewhat2: Quite a lot3: Very much

* Obligatoria

1. DNI *

2. Do you feel dizzy now? *

- ◯ YES
- ◯ NO

3. General discomfort *

- ◯ 0
- ◯ 1
- ◯ 2
- ◯ 3

4. Fatigue *

- ◯ 0
- ◯ 1
- ◯ 2
- ◯ 3

5. Headache *

- ◯ 0
- ◯ 1
- ◯ 2
- ◯ 3

6. Eye strain *

- ◯ 0
- ◯ 1
- ◯ 2
- ◯ 3

7. Difficulty focusing *

- ◯ 0
- ◯ 1
- ◯ 2
- ◯ 3

8. Increased salivation *

- ◯ 0
- ◯ 1
- ◯ 2
- ◯ 3

9. Sweating *

- ◯ 0
- ◯ 1
- ◯ 2
- ◯ 3

10. Nausea *

- ◯ 0
- ◯ 1
- ◯ 2
- ◯ 3

11. Difficulty concentrating *

- 0
- 1
- 2
- 3

12. Fullness of head *

- 0
- 1
- 2
- 3

13. Blurred vision *

- 0
- 1
- 2
- 3

14. Dizzy (eyes open) *

- 0
- 1
- 2
- 3

15. Dizzy (eyes closed) *

- 0
- 1
- 2
- 3

16. Vertigo *

- ◯ 0
- ◯ 1
- ◯ 2
- ◯ 3

17. Stomach awareness *

- ◯ 0
- ◯ 1
- ◯ 2
- ◯ 3

18. Burping *

- ◯ 0
- ◯ 1
- ◯ 2
- ◯ 3

Este contenido no está creado ni respaldado por Microsoft. Los datos que envíe se enviarán al propietario del formulario.

Microsoft Forms
