# Supplementary material for: Measuring Stress and Perceptions for a Virtual Reality–Based Pericardiocentesis Procedure Simulation for Medical Training: Usability Study
Source: JMIR Serious Games. 2025 May 7;13:e68515. doi: 10.2196/68515 (PMC12303137; doi:10.2196/68515)
Supplement: Multimedia Appendix 5 [file games-v13-e68515-s005.pdf]

Multimedia Appendix 5

ID	rMSSD (ms)	LF/HF Ratio	SD1/SD2 Ratio	Poincaré Area	SUS	SUS Usability	SUS Learning	PQ	PQ Naturalness	SSQ	PSSUQ
1	188	2,7	0,89	63566	95	20	18	116	13	26,16	1,63
2	466	1,9	0,85	400176	90	19	17	119	10	0	1,38
3	180	0,9	0,81	62640	85	18	16	112	19	0	2,75
4	192	1,1	0,94	61952	82,5	15	18	122	12	5,71	2,25
5	402	0,9	0,83	308001	87,5	20	15	109	10	7,17	1,38
6	230	0,5	0,89	93710	52,5	12	9	106	17	46,46	4,69
7	207	0,5	1,04	65577	75	16	14	114	14	12,87	5,31
8	299	0,7	0,92	153184	80	15	17	110	15	56,31	3,94
9	214	0,6	1,01	72105	42,5	9	8	113	15	8,89	5,19
10	260	0,7	0,89	119078	100	20	20	114	10	0	1
11	192	0,6	0,91	63661	87,5	17	18	113	11	0	6
12	352	0,3	0,92	211209	77,5	16	15	125	15	0	6,5
13	456	0,4	0,9	365304	72,5	16	13	88	16	0	2,44
14	487	1,5	0,87	426879	65	16	10	103	12	0	1,13
15	344	0,8	0,88	210700	95	20	18	114	14	0	6,13
16	227	0,5	0,79	103688	70	13	15	90	17	3,18	3,19
17	506	1,8	0,82	490364	67,5	12	15	112	16	0	3
18	276	1,6	0,78	152540	90	19	17	103	14	3,18	2,5
19	91	0,6	0,64	20624	80	17	15	112	10	11,41	1,31
20	322	0,7	0,97	167610	75	14	16	98	19	0	2,06
21	384	0,5	0,95	244390	90	18	18	123	14	3,18	1,63
22	246	1,3	0,76	125726	40	8	8	108	14	11,41	1,81
23	171	2,2	0,83	55119	77,5	19	12	107	18	0	1,94
24	180	0,9	0,81	62640	87,5	18	17	106	11	2,53	1,56
25	396	0,9	0,99	250711	75	16	14	103	12	0	1,81
26	408	1,6	0,88	298705	97,5	19	20	106	15	0	6,38
27	128	1,3	0,78	33448	70	17	11	109	15	6,36	2,5
28	237	0,8	0,97	91835	92,5	19	18	134	18	20,45	6,88
29	420	0,5	0,83	337030	80	18	14	98	9	7,58	1,56
30	355	0,7	0,9	220002	80	19	13	119	13	21,11	6,63
31	320	0,6	0,98	165448	70	17	11	120	14	2,53	3,19
32	254	0,5	0,95	107442	52,5	12	9	94	14	15,81	3,75
33	185	0,6	0,9	60086	80	19	13	108	12	3,18	5,88
34	304	0,8	0,82	177641	90	19	17	114	11	31,21	1,13
35	305	1,1	0,82	179824	62,5	11	14	107	16	64,37	1,63
36	299	0,7	0,92	153184	65	15	11	104	15	34,39	5,5
37	244	0,5	1,09	86415	62,5	14	11	100	17	0	1,5
38	319	0,3	0,85	188859	87,5	17	18	98	11	26,81	2,38
39	351	1,2	0,91	213556	95	20	18	118	21	43,02	1
40	297	0,6	0,9	154377	75	14	16	120	14	0	1,69
41	261	0,5	0,83	130187	77,5	14	17	110	14	0	2,06
42	269	0,9	0,73	156611	67,5	15	12	120	14	2,53	2,19
43	127	1	0,65	39301	15	0	6	108	16	19,8	1,94

44	371	0,9	0,99	220605	75	14	16	92	15	51,67	5,13
45	197	0,4	0,91	66375	55	11	11	119	17	13,94	3,31
46	338	0,7	0,87	206481	67,5	11	16	111	12	52,56	2,13
47	224	0,7	0,95	83418	52,5	15	6	116	14	25,75	1,94
48	435	0,7	0,9	330922	65	13	13	103	17	10,76	2,44
49	197	0,6	0,9	68172	60	13	11	99	13	10,76	4,75
50	294	0,6	0,86	158788	87,5	19	16	112	14	7,17	1,38
51	420	1	0,87	320037	67,5	20	7	98	9	31,06	6,81
52	337	0,8	0,88	204228	95	20	18	100	8	20,3	1
53	391	0,7	0,81	297615	75	16	14	105	11	35,44	2,44
54	350	0,6	0,76	253212	75	16	14	120	15	0	1
55	234	0,6	0,86	100650	65	14	12	108	12	0	2,63
56	217	0,9	0,82	89403	87,5	20	15	102	15	12,87	6,75
57	456	0,9	0,86	382554	60	13	11	98	18	0	3,94
58	132	1,6	0,63	44296	70	14	14	118	18	21,11	2,5
59	333	0,6	0,73	238735	72,5	14	15	98	14	46,61	2,75
60	246	0,3	1,04	91835	57,5	15	8	97	14	0	4,81
61	314	0,8	0,9	172266	17,5	1	6	101	14	12,72	1,75
62	472	0,8	0,82	428111	77,5	20	11	101	14	17,12	1,19
63	250	0,6	0,88	111768	100	20	20	119	13	0	1,63
64	306	0,9	0,85	173840	87,5	18	17	126	10	9,54	1,94
65	402	0,8	0,72	351531	62,5	17	8	97	17	9,69	5,81
66	306	0,9	0,83	190003	85	17	17	125	15	0	6,13
67	216	0,5	0,99	74022	80	15	17	125	13	13,94	5,31
68	352	0,6	0,81	240935	55	12	10	93	15	19,39	2,5
69	259	0,2	1,02	103484	82,5	18	15	109	12	0	1,38
70	241	0,4	0,9	102070	80	17	15	120	15	0	5,81
71	323	0,9	0,89	184172	85	18	16	121	13	15,4	1,31
72	318	1,2	0,85	186610	77,5	15	16	101	7	6,36	2,13
73	296	1	0,81	170211	50	20	0	96	12	5,05	6,63
74	328	0,7	0,82	206993	75	15	15	102	8	38,23	1,19
75	356	0,6	0,86	231962	80	18	14	101	14	18,99	1,5
76	292	0,6	0,91	148270	77,5	16	15	107	12	60,53	1,63
77	379	0,9	0,8	282894	47,5	9	10	104	18	50,29	4,31
78	389	0,8	0,9	284699	80	15	17	96	16	0	2,31
79	53	2,1	0,32	14325	85	18	16	110	15	0	5,88
80	245	0,6	0,97	97848	82,5	15	18	109	13	0	1,5
81	228	0,9	0,84	96607	90	19	17	114	10	0	1,44
82	197	0,7	0,83	73362	82,5	20	13	114	10	20,45	1,06
83	254	0,8	0,86	118752	72,5	14	15	103	12	26,16	2,19
84	140	1,4	0,64	48207	82,5	18	15	120	18	0	2,06
85	198	0,9	0,88	69931	82,5	20	13	117	12	0	1,19
86	225	0,4	0,97	82938	40	7	9	102	19	15,4	4,69
87	173	0,6	1,04	45597	80	17	15	108	12	10,35	2,13
88	218	0,4	0,97	77911	70	16	12	111	12	2,53	1,81
89	297	0,6	0,85	162954	77,5	19	12	116	12	0	1,38

90	238	0,7	0,79	113087	87,5	20	15	108	12	10,76	6,44
91	39	3	0,47	5277	82,5	15	18	110	12	10,76	2,44
92	220	1,1	0,81	94587	42,5	7	10	102	14	9,69	3,25
93	243	0,6	0,86	108611	72,5	15	14	102	19	2,53	2,56
94	569	1,1	0,9	567195	67,5	19	8	100	14	13,29	1,69
95	336	1,5	0,99	179447	87,5	18	17	117	15	0	5,88
96	169	1,7	0,75	60318	82,5	18	15	120	14	0	1,88
97	123	1,4	0,74	32898	42,5	8	9	101	13	3,18	2,25
98	413	1,6	0,78	342169	97,5	19	20	114	14	8,23	1,13
99	324	1	0,81	202158	75	14	16	106	14	0	5,88
100	270	0,7	0,92	124809	52,5	12	9	120	12	11,41	4,44
101	274	0,5	0,84	141396	50	20	0	105	14	11,41	1
102	149	0,9	0,76	46621	95	18	20	119	13	3,18	1,25
103	110	1,6	0,71	26954	90	18	18	122	20	0	1,56
104	238	1,3	0,7	126669	75	15	15	107	14	0	3
105	420	0,6	0,91	303242	87,5	19	16	106	9	17,51	1
106	203	0,4	0,86	76001	72,5	18	11	113	17	35,05	1,81
107	244	0,9	0,87	108155	95	20	18	108	7	19,65	1,44
108	229	0,6	0,88	94153	85	19	15	99	14	34,89	1,88
109	222	0,7	0,85	92325	80	18	14	119	10	24,29	1,81
110	226	0,8	0,87	92488	75	15	15	110	11	0	1,63
111	335	1,2	0,79	224111	57,5	12	11	96	19	0	2,13
112	312	0,6	0,91	169407	95	20	18	126	11	5,05	1,38
113	191	0,4	1	57255	72,5	19	10	114	13	0	1,13
114	383	0,6	0,86	269033	90	19	17	117	13	9,69	1,31
115	233	1,3	0,79	108856	82,5	17	16	119	8	7,17	2,13
116	279	0,5	0,9	134918	100	20	20	118	14	10,35	1,31
117	479	0,5	0,82	437714	80	16	16	108	16	37,81	5,94
118	202	1	0,92	70082	80	17	15	123	18	0	6,31
119	248	0,9	0,83	116666	80	18	14	106	12	3,18	5,75
